# “It is natural so it must be safe!”: Cannabis use during pregnancy, an update

**DOI:** 10.1192/j.eurpsy.2021.2201

**Published:** 2021-08-13

**Authors:** S. Freitas Ramos, D. Cruz E Sousa, B. Jesus, J. Martins Correia, M.I. Fonseca Marinho Vaz Soares, J. Mendes

**Affiliations:** Department Of Psychiatry And Mental Health, Local Health Unit of Guarda, Guarda, Portugal

**Keywords:** Cannabis, pregnancy

## Abstract

**Introduction:**

Rates of cannabis use among pregnant women have been increasing. Psychiatrists may be required to provide counselling regarding marijuana use in pregnancy for their patients.

**Objectives:**

To produce an up-to-date review of cannabis effects on pregnancy and the offspring.

**Methods:**

We performed a non-systematic review of the literature apropos a clinical case.

**Results:**

A 31-years-old, 22-weeks pregnant woman presented with severe anxiety, panic attacks and insomnia which she managed solely with cannabis. She had been previously treated with antidepressants and benzodiazepines with symptom remission but had suspended before her pregnancy without medical advice. She believed medication was more harmful to the baby than her cannabis use. There is little perception of risk concerning cannabis use in pregnant woman. Information on cannabis use is less likely to be obtained from healthcare providers than from anecdotal experiences, Internet searching and advice from friends and family. Prenatal use of cannabis has been associated with anaemia in the mother, whereas in the offspring it is associated with reduction in birth weight and greater likelihood of placement in intensive care units. There is insufficient evidence to support an association between marijuana use and any specific congenital abnormality, but also to demonstrate its safety.

**Conclusions:**

It is essential for psychiatrists to have up-to-date knowledge of the effects of cannabis on the pregnancy and the offspring to properly counsel their patients. However, the effects of cannabis on maternal and foetal outcomes remain generally unknown. With rising numbers of female users, there is urgent need for further research.

**Disclosure:**

No significant relationships.

